# Osteopathic Acceptance in General Surgery Residency: A Five-Year Review of Resident Outcomes

**DOI:** 10.7759/cureus.98587

**Published:** 2025-12-06

**Authors:** Samuel M Baule, Justin Nguyen, Donna Mehdiyar, Mia A Panlilio, Hayden Flume, Allen Hanna, Aila Cordero, Julia Shepherd, Ryan Johnson, Julieanne P Sees

**Affiliations:** 1 College of Osteopathic Medicine, Marian University, Indianapolis, USA; 2 College of Osteopathic Medicine, Edward Via College of Osteopathic Medicine, Monroe, USA; 3 College of Osteopathic Medicine, Midwestern University, Downers Grove, USA; 4 Research and Scholarly Activity, Rocky Vista University College of Osteopathic Medicine, Parker, USA; 5 College of Osteopathic Medicine, Texas College of Osteopathic Medicine, Fort Worth, USA; 6 College of Osteopathic Medicine, Arkansas College of Osteopathic Medicine, Fort Smith, USA; 7 Dr. Kiran C. Patel College of Osteopathic Medicine, Nova Southeastern University, Fort Lauderdale, USA; 8 College of Osteopathic Medicine, University of New England, Portland, USA; 9 Biostatistics, Capitol Technology University, Laurel, USA; 10 Orthopedics, American Osteopathic Association, Chicago, USA; 11 Haub School of Business, Saint Joseph's University, Philadelphia, USA

**Keywords:** general surgery residency, osteopathic medical graduate, osteopathic medical students, residency application, resident demographics

## Abstract

Introduction: A well-trained and equitably distributed general surgery workforce is an essential component to meeting national healthcare needs. The composition of surgical residency programs is under continuous evaluation as it can shape the future of surgically trained doctors. While prior studies have highlighted trends such as increasing presence of international medical graduates in general surgery, there is still limited data on the osteopathic representation and how its trends may relate to resident demographics. The key interests include integration of osteopathic graduates, where disparities persist despite ongoing shifts. Systemic changes, such as the recent transition to a single accreditation system, may have the potential to influence match rates in other surgical specialties. This study offers a contemporary, nationwide analysis of 2024-2025 general surgery residency data, which highlights growing and changing trends of surgical resident demographics and program characteristics to better understand trends in matching into general surgery.

Methods: This study encapsulates publicly available data primarily extrapolated from the National Residency Match Program (NRMP) Main Residency Match data and using the Fellowship and Residency Electronic Interactive Database Access (FREIDA), maintained by the American Medical Association (AMA). FREIDA was used to locate residency program websites, serving as the primary source for detailed resident and faculty information and where more extensive data collection was conducted. Programs were excluded if essential residency data were not publicly available. Other exclusion criteria include preliminary positions and the absence of critical data relevant to categories of interest. Data collection was obtained and organized into different domains that included categorical data among resident and program data (e.g., Medical Doctor (MD) vs Doctor of Osteopathic Medicine (DO), American Osteopathic Association (AOA) affiliation, Program Setting, Gender, and Postgraduate Year). Descriptive statistics were used to summarize program size, resident demographics, and degree. Chi-square tests were applied to evaluate proportional differences across programs.

Results: Of the 364 Accreditation Council for Graduate Medical Education (ACGME) accredited general surgery residency programs identified, 292 met inclusion for this analysis. In total, 7,846 residents across these programs were evaluated. Among the 292 programs, 103 (35.3%) demonstrated equal to or greater-than-expected representation of DO residents compared to the national average of 14.3% calculated from NRMP data. In contrast, 189 programs (64.7%) have below-average DO representation. Forty programs (13.7%) were classified as DO dominant, while 246 programs (84.2%) had more MDs than DOs. Of the historically osteopathic (AOA affiliated) programs, 32 (88.9%) demonstrated above-average DO representation relative to NRMP benchmarks. DOs represented 13.8% (n=1082) of the total 7,846 residents analyzed.

Conclusion: Osteopathic medical students continue to face challenges despite progress in DO representation in general surgery. This study aims to highlight the current landscape of osteopathic inclusion, underscore the potential to further grow DO interest in general surgery, and emphasize the ongoing efforts needed to promote equitable representation across training programs for integration of osteopathic physicians into the surgical workforce.

## Introduction

Having a well-trained and equitably distributed general surgery workforce is crucial for meeting national healthcare demands. This is true in surgical fields, where demands often exceed the supply of professionals. Following conservative projections through 2034, the United States (US) is expected to have a shortage of over 15,000 surgeons [1]. The composition of surgical residency programs, encompassing resident demographics and training, is a central focus of this evaluation, as these factors shape the future specialty landscape. Previous studies have documented evolving trends in residency match outcomes, including the growing presence of international medical graduates in general surgery training [2]. The goal of this investigation is to assess the integration of osteopathic graduates into the current surgical residency landscape.

There remains a relative lack of literature examining the representation of graduates from osteopathic medical schools (DOs) in general surgery. Historically, osteopathic physicians have had a minority stake within the surgical community, with current estimates below 9% of the larger surgical population [3]. While the osteopathic surgical community is small, a 30-year match trend analysis found that US DO applicants have been steadily increasing in match rate by about 0.4% each year into general surgery [4]. This may seem like a small number; however, it points to a trend of increasing osteopathic presence within the surgical community and the medical community at large. Currently, 25% of all medical students are training to become osteopathic physicians and surgeons [5]. Given the historically increased match rates and the growing number of medical graduates, further investigation is needed to understand why fewer osteopathic medical students are entering surgical residencies. The 2020 transition to a single graduate medical education (GME) accreditation system has further altered the residency training environment. Evidence suggests that this reform reduced overall match rates for osteopathic graduates, particularly in certain surgical fields such as general surgery [6]. These shifts highlight an important, yet underexplored, demographic trend within the surgical workforce.

While existing studies have examined these dynamics in narrow contexts, such as single-specialty reviews or the broader implications of accreditation changes, few studies have developed a comprehensive, contemporary analysis of osteopathic representation in general surgery. Using data from the 2024-2025 academic year, this study addresses this gap by examining the interplay between categorical general surgery resident demographics and program characteristics across the United States. With this integrated approach, our research offers an updated understanding of the factors shaping the current landscape of general surgery training in the U.S. This study aims to characterize the current representation of osteopathic medical graduates in general surgery residency programs and to examine trends over the past five years in order to better understand factors influencing osteopathic participation in surgical training.

## Materials and methods

Data collection focused on resident data including name, degree type (DO/MD), medical school attended, residency program, postgraduate year (PGY) and American Osteopathic Association (AOA) status. 

Data was primarily obtained using the Fellowship and Residency Electronic Interactive Database Access (FREIDA), maintained by the American Medical Association. FREIDA was used to locate residency program websites, which served as the primary source for detailed resident and faculty information. Programs were excluded if essential residency data were not publicly available, which applied primarily to military programs.

Eight authors participated in data acquisition. Programs were divided equally among the authors, and each dataset was independently verified by a second author. All information was compiled and organized in a centralized Google Sheet. Discrepancies were resolved collectively, defaulting to more conservative or clearly verifiable figures. 

Data collection ranged from October 2024 through January 2025. Group consensus identified inconsistencies in the reporting of academic ranks across program websites, resulting in the halting of gathering detailed faculty rank data. 

Google Sheets was used for data organization and descriptive statistics. Inferential analyses were carried out in Microsoft Excel (version 16.101.3; Microsoft Corporation, Redmond, USA), with built-in functions used for chi-square tests and a manually implemented formula applied for Fisher’s exact test. All statistical results, including chi-square and Fisher’s exact test outputs, were independently verified using reputable online tools, including GraphPad QuickCalcs (GraphPad Software, San Diego, CA) and the Standard Deviation Calculator chi-square tool. Chi-square tests were conducted to determine associations between degree types, PGY levels, and DO representation relative to the national average. Fisher’s exact test was used in place of the chi-square test when expected cell counts were less than 5, as in the case for analysis of AOA-affiliated programs and below-average DO representation. Historically, AOA-affiliated programs were identified through the AOA website, and their ACGME identification numbers were cross-referenced with our dataset to determine which programs held this designation.

A logistic regression analysis was obtained to evaluate the association between program characteristics and the likelihood of a resident being a DO. Predictor variables included PGY level, AOA affiliation, and program size. Program size is defined as the total number of residents within each program across all PGY levels. For each predictor, odds ratios (ORs), 95% confidence intervals (CIs), and p-values were reported regardless of statistical significance to provide a complete overview of the model's outputs.

## Results

**Figure 1:**
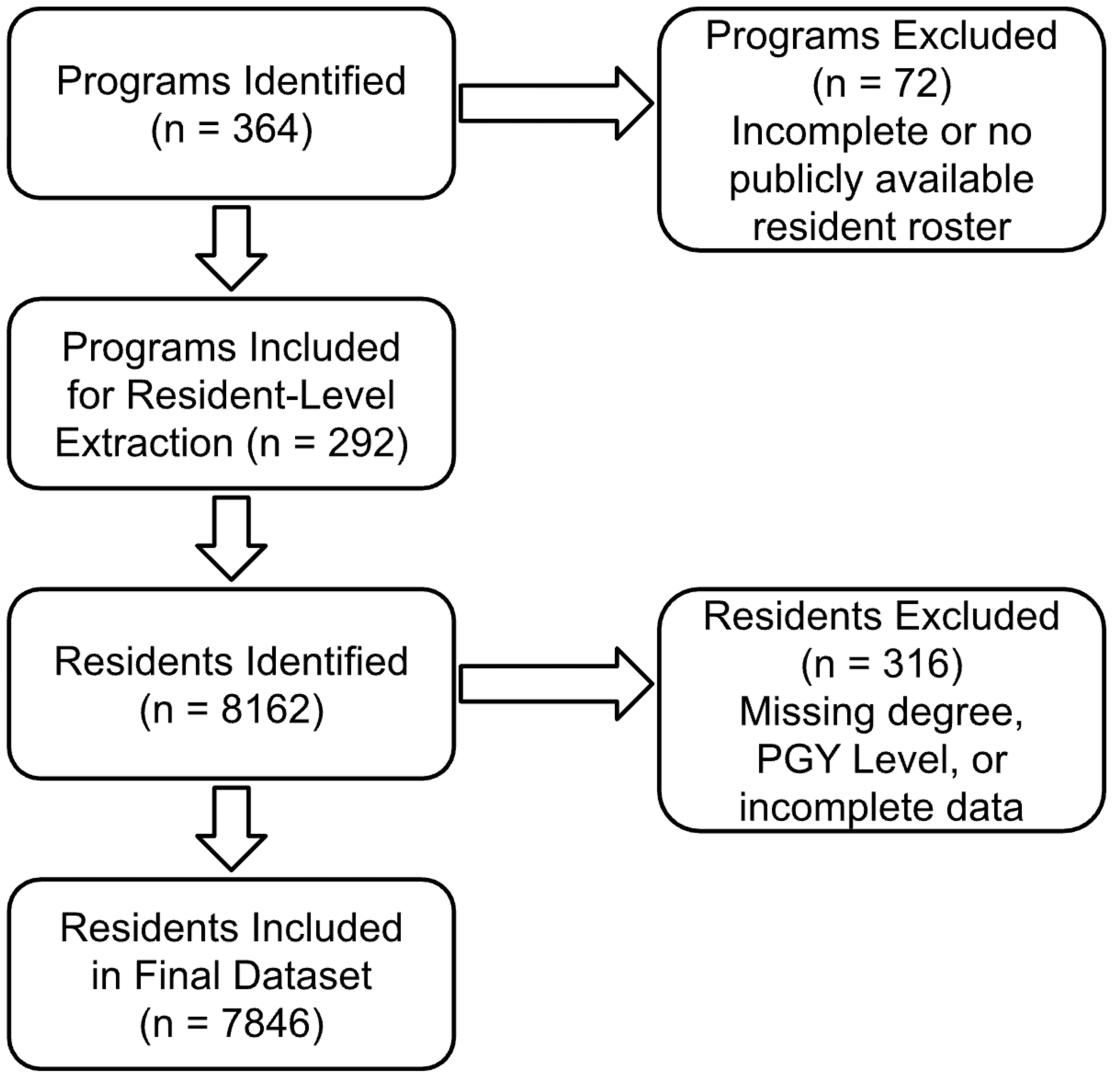
Flow Diagram of Program and Resident Inclusion for the 2024–2025 ACGME-Accredited General Surgery Dataset Flow diagram depicting the identification, screening, and inclusion of ACGME-accredited general surgery residency programs and residents for analysis. This is a visual depiction of the process outlined in the above paragraph. ACGME: Accreditation Council for Graduate Medical Education

A total of 364 general surgery residency programs from the list of ACGME-accredited programs for the 2024-2025 academic year. This study included 292 of the 364 residency programs based on publicly available residency data for their corresponding residents. The study identified a total of 8162 residents through 364 programs. Three hundred and sixteen of those residents were removed from the data pool as information regarding degree type could not be identified from publicly available resources. The final analysis included a total of 292 programs and 7846 residents. Figure 1 displays a flow diagram of the inclusion and exclusion process.

Information based on PGY was obtained and stratified by PGY, and degree. Table 1 displays these results by number and as proportions.

**Table 1:** Percent Distribution of DO and MD Residents Across PGY Years in General Surgery Programs "%" indicates the percentage of total residents (N=7,846) within each category. PGY: Postgraduate year; MD: Medical doctor; DO: Doctor of osteopathic medicine

PGY Year	Resident Grad Year	% of Residents	% of DOs	% of MDs
PGY1	2029	23.54% (1847)	15.43% (285)	84.57% (1562)
PGY2	2028	20.90% (1640)	13.72% (225)	86.28% (1415)
PGY3	2027	19.59% (1537)	13.40% (206)	86.60% (1331)
PGY4	2026	18.44% (1447)	12.79% (185)	87.21% (1262)
PGY5	2025	17.52% (1375)	13.16% (181)	86.84% (1194)
(χ²) = 6.06, df = 4, p-value = 0.195				

Of the 292 general surgery residency programs evaluated, 40 (13.70%) had a higher number of osteopathic residents than allopathic residents, while an additional six programs (2.05%) had equal representation. To determine whether programs were above or below the national average for DO representation, we used National Residency Match Program (NRMP) data. Across the 2020-2024 match cycles, 1159 of 8093 matched general surgery applicants were osteopathic graduates, corresponding to a national average of 14.3%. Using this benchmark, 189 programs (64.73%) were identified as having below-average osteopathic representation. Tables 2, 3 display the results of this analysis. 

**Table 2:** Comparison of General Surgery Programs by DO Representation Relative to the National Average The number "#" and percentage of general surgery residency programs classified as above or below the national average for DO representation. The symbol “#” denotes the count of programs within each category. A chi-square test (χ² = 25.33, df = 1, p < 0.000001) demonstrated a statistically significant difference between groups. DO: Doctor of osteopathic medicine

	# of Programs	% of Total Programs
Above Average	103	35.27%
Below Average	189	64.73%
Total	292	`
(χ²) = 25.33, df = 1, p-value < 0.000001		

**Table 3:** Distribution of General Surgery Residency Programs by Degree Type Dominance (DO vs MD) The symbol “#” denotes the count of programs within each category. DO or MD Dominant Programs indicate that a program has a majority of that category of resident type. DO Dominant Programs are programs with more DO than MD residents. MD Dominant Programs are programs with more MD than DO residents. MD: Medical doctor; DO: Doctor of osteopathic medicine

	# of Programs	% of Total Programs
MD Dominant Programs	246	84.25%
DO Dominant Programs	40	13.70%
Programs with Equal #	6	2.05%
Total	292	100%

Residency programs that were identified as being historically AOA-affiliated numbered 36. The vast majority of these programs had above-average representation of osteopathic trainees, with 32 of the programs exceeding the NRMP average number of DOs. Table 4 displays these results. 

**Table 4:** Comparison of AOA-Affiliated and Non-AOA Programs by DO Representation Relative to the National Average "≥ Avg DO" indicates that a program has above or equal to national average representation of DO residents. "< Avg DO" indicates that a program has below national average representation of DO residents. "AOA-affiliated" indicates that a program was historically affiliated with the American Osteopathic Association for accreditation. "Non-AOA" indicates that a program was not historically affiliated with the American Osteopathic Association for accreditation. AOA: American Osteopathic Association

	≥ Avg DO	< Avg DO	Total
AOA-affiliated	32	4	36
Non-AOA	71	185	256
Total	103	189	292
(χ²) = 49.05, df =1, p-value = < 0.000001			

Fisher’s exact test was used to assess the association between AOA affiliation and having ≥ average DO representation among general surgery programs. The result was statistically significant (two-tailed p < 0.001), indicating that AOA-affiliated programs were more likely to have ≥ average DO representation compared to non-AOA programs.

The results from a logistic regression for the outcome of DO with the predictors of PGY, AOA affiliation of residency program and size of program are included in Table 5. 

**Table 5:** Logistic Regression Analysis of Predictors for an Individual Resident Being a DO AOA: American Osteopathic Association; PGY: Postgraduate year; DO: Doctor of osteopathic medicine

Predictor	Odds Ratio (OR)	95% CI	p-value
PGY	0.91	0.86 – 0.96	0.0003
AOA Affiliation	9.6	7.94 – 11.62	<0.0001
Program Size	0.95	0.94 – 0.96	<0.0001
Intercept	0.64	0.50 – 0.82	0.0005

A simple linear regression was conducted to evaluate the relationship between program size and the proportion of DO residents. Results indicated a statistically significant inverse association between the number of total residents and the percentage of DOs (β = -0.0086, SE = 0.0010, t(289) = -8.33, p < 0.001). The overall model was significant, F(1, 289) = 69.39, p < 0.001, and explained approximately 19.4% of the variance in DO representation (R² = 0.194). This suggests that, on average, for each additional resident in a program, the percentage of DOs decreases by 0.86 percentage points.

## Discussion

A chi-squared test for proportionality did not identify statistically significant differences in the number of DO residents across individual PGY levels (χ² = 6.06, df = 4, p = 0.195), indicating that year-to-year variation in DO representation did not exceed what would be expected by chance. However, when PGY was treated as an ordinal variable in a logistic regression model, advancing PGY level was found to be a statistically significant negative predictor of DO status (OR = 0.91 per PGY year, 95% CI 0.86-0.96, p = 0.0003). This suggests that, while no single PGY cohort differed significantly from the others in isolation, there is a significant overall decline in DO representation as trainees progress from PGY1 to PGY5. This pattern may reflect structural or attritional factors within surgical training, and future longitudinal analyses will be necessary to determine whether the higher proportion of DOs observed in more recent entering cohorts translates into sustained growth in osteopathic representation across all PGY levels over time.

Currently, only 11% of practicing physicians in the United States are osteopathic graduates [7]. Estimates of current osteopathic medical students far exceed that number and continue to grow, ranging from 25% to 30% of all medical students aspiring for an osteopathic medical degree [5,8]. This publicly available data and the trend shown within this project emphasize the importance of further exploration of osteopathic representation as the future matriculants enter residency. It is important to identify that while DO general surgeons make up a minority of the practicing field, data support that they have at least equivalent training. For example, an investigation into patient outcomes stratified by degree type saw no statistically significant difference in post-surgical complications [3]. Additionally, in a comparison of training metrics, an investigation stratified by degree type saw no statistically significant differences in American Board of Surgery In-Training Examination (ABSITE) performance between the two degrees [9]. 

There was a strong and highly significant association between historical AOA affiliation and the likelihood of a program exceeding the national NRMP average for DO representation. Programs with AOA heritage were far more likely to train a disproportionate number of osteopathic residents (χ² = 49.06, df = 1, p < 0.000001). This conclusion is reinforced by a Fisher’s exact test (two-tailed p < 0.001), again highlighting that AOA-affiliated programs were more likely to have ≥ average DO representation. This pattern is expected, as many of these programs have a long tradition of recruiting and training osteopathic candidates, with the earliest AOA-accredited general surgery residencies dating back to 1948 [10]. In addition, these programs were likely more adept at evaluating COMLEX-USA scores relative to MD-dominant programs. This is particularly relevant given recent evidence demonstrating that COMLEX performance strongly predicts outcomes on the ABSITE, with Level 1 and Level 2-CE scores showing moderate to strong correlations with ABSITE scores (r = 0.42-0.51) and a Level 2-CE score of 550 associated with a 91% predicted probability of passing the ABS qualifying exam [11].

Additionally, when examining DO distribution nationally, we observed a statistically significant imbalance: far fewer programs than expected met or exceeded the national DO representation average of 14.32% (χ² = 25.33, p < 0.000001). The average, as calculated from NMRP data of matched DOs and matched applicants from 2020-2024, shows that nationally, osteopathic graduates are overperforming. Data from the AOA reports that only about 4% of osteopathic graduates pursue general surgery [7]. The data suggests that DOs are being overrepresented in AOA programs and significantly underrepresented in non-AOA-affiliated residency programs. While this data does not identify individual programs as the cause for this culture, it provides insight into a pattern that could be interpreted as indicative of bias against osteopathic trainees. This finding underscores the inequitable distribution of DO trainees across general surgery programs. 

Program size also emerged as a significant predictor of DO representation. Larger programs were less likely to include osteopathic residents, with a statistically significant negative correlation between program size and percentage of DOs. Specifically, for every one-resident increase in program size, the odds of an individual being a DO trainee decreased by 4.9% (OR = 0.95, CI = 0.94-0.96, p < 0.0001). This supports a common trend in osteopathic medicine, as most osteopathic medical schools were founded with goals of supporting underserved communities in primary care [12]. This emphasis increases the likelihood that graduates from these institutions pursue practice in underserved communities, regardless of their eventual specialty choice. Additionally, smaller programs often operate with more limited funding and consequently place less emphasis on research, a pattern consistent with the broader trend that DO schools typically maintain more modest research infrastructures, reflective of systemic underfunding. For instance, despite educating one-quarter of U.S. medical students, osteopathic schools receive only 0.1% of NIH funding [13,14]. They submit just 0.4% of NIH grant applications, with MD schools enjoying significantly higher success rates [14]. This reflects another study identifying a clear degree disparity in research production [15]. Additionally, DO-granting institutions generally feature more modest research programs and fewer scholarly opportunities for students, which gives credence to the idea that students from these institutions would pursue GME in a similar pattern. 

Logistic regression analysis revealed that three factors including historical AOA affiliation of residency, smaller program size, and more recent PGY year were all significant positive predictors of being a DO resident. Each increase in PGY year (i.e., from PGY-1 to PGY-2) was associated with a 9% decrease in the odds of being a DO (OR = 0.90, CI = 0.86-0.96, p = 0.0003), suggesting a growing proportion of osteopathic trainees in the most recent match cohorts. 

Residents in historically AOA-affiliated programs were 9.6 times more likely to be osteopathic trainees compared to those in non-affiliated programs (OR = 9.6, CI = 7.94-11.62, p < 0.0001), reinforcing the strong correlation between program heritage and DO representation.

While this study identified significant predictors of DO representation within general surgery residency programs, several limitations must be acknowledged. First, the observational nature of the study precludes causal inference; the findings reflect associations and should not be interpreted as evidence of direct causation. Second, the analysis relied on publicly available data from residency program websites, which may be subject to inaccuracies, delays in updates, or variability in the completeness of resident profiles. Although programs without full resident information were excluded and care was taken to ensure accuracy, the validity of the dataset ultimately depends on the accuracy of reported program information. Additionally, 312 residents with incomplete profiles were excluded from the final analysis. While this represents a small proportion of the total resident population, the exclusion could introduce bias if the missing data are not randomly distributed. For example, if a substantial subset of excluded residents were graduates of osteopathic medical schools, the analysis may underestimate the true proportion of DO trainees in general surgery. Finally, this cross-sectional analysis reflects the current resident cohort and may not fully capture longitudinal trends in osteopathic representation. Future research incorporating longitudinal data, direct program reporting, and multiyear trend analysis would strengthen the generalizability and depth of these findings.

## Conclusions

This study continues to highlight the persistent underrepresentation of osteopathic graduates participating in general surgery residency programs. These results point to imbalances in how osteopathic graduates are represented within general surgery residency programs. Addressing these disparities may require focused review by accrediting bodies, surgical education organizations, and residency programs, with attention to recruitment, selection processes, and transparency in applicant criteria. While these disparities may be due to individual differences in residency choice, our data suggest that more analysis needs to be completed on additional factors such as geographic distribution, gender, and program characteristics to make that conclusion. With the number of osteopathic medical students steadily rising, it will become increasingly important to foster inclusive training environments that reflect the future makeup of the physician workforce. 
